# Diabetes mellitus and bone health: epidemiology, etiology and implications for fracture risk stratification

**DOI:** 10.1186/s40842-018-0060-9

**Published:** 2018-04-25

**Authors:** Rodrigo J. Valderrábano, Maria I. Linares

**Affiliations:** University of Miami Miller School of Medicine, Dominion Tower 1400 NW 10th Ave, Ste. 805A, Miami, FL 33136 USA

**Keywords:** Diabetes mellitus, Type 1 diabetes mellitus, Type 2 diabetes mellitus, Bone mineral density, Fracture, Fracture risk stratification, Osteoporosis

## Abstract

Skeletal fractures can result when there are co-morbid conditions that negatively impact bone strength. Fractures represent an important source of morbidity and mortality, especially in older populations. Diabetes mellitus is a metabolic disorder that has reached worldwide epidemic proportions and is increasingly being recognized as a risk factor for fracture. Type 1 and Type 2 diabetes have different effects on bone mineral density but share common pathways, which lead to bone fragility. In this review, we discuss the available data on diabetes and fractures, bone density and the clinical implications for fracture risk stratification in current practice.

## Background

Osteoporosis is a systemic disease, which confers decreased bone strength and increased fracture risk [1–3]. Hip fractures especially are a major source of morbidity and mortality in older populations [4] and present an increasing public health burden [5]. Diabetes mellitus type 2 accounts for 90–95% of the incidence of diabetes [6, 7] and its prevalence is increasing worldwide [8]. Type 2 diabetes mellitus (T2DM) is associated with an increased bone mineral density (BMD) but a paradoxically increased risk for skeletal fractures [9–11]. Type 1 diabetes mellitus (T1DM) is less prevalent, but its incidence is rising, especially in the very young [12] and it has also been associated with increased fracture risk [13]. The causative mechanisms for this association are the subject of study by several groups but have not been completely elucidated. This article aims to evaluate the relationship of diabetes etiology, duration and glucose control with BMD and skeletal fracture. We also discuss potential strategies that increase the accuracy of fracture risk estimates in populations with diabetes mellitus, which could be applied to current clinical practice.

### Diabetes and fracture risk

Diabetes mellitus has been associated with increased fracture risk by several groups. The Nurses’ Health Study followed 109,983 women aged 34–59 years old with biennial questionnaires for over 20 years and monitored the occurrence of hip fractures. They found that the risk of hip fracture in women with T1DM was six-fold higher compared with those without diabetes [14]. The Health Improvement Network (THIN) study used longitudinal electronic medical record data in the United Kingdom (UK) to evaluate incident fractures in men and women with T1DM from age 0 to 89 years old, across a median of 4.7 years of follow up. They found that the risk of incident fracture of any type increased in both sexes and in all age groups compared to those without diabetes. When stratified by age, women with T1DM ages 40–49 had the highest risk for fracture at any site, 82% higher than women without diabetes after multivariate adjustment. Men aged 60–69 with T1DM also had double the risk of fracture at any site compared to men without diabetes in the same age group [15]. The same study performed secondary analyses evaluating hip fracture specifically and found that men aged 60–69 with T1DM had 421% increased risk of hip fracture as compared to those without diabetes after adjustment for confounders. Young women with T1DM, in the 30–39 years old group had a 316% increase in the risk of hip fracture compared to those without, the highest magnitude increase in this sex. The authors concluded that fracture risk in T1DM is increased early on in life and that it is maintained throughout. The magnitude of fracture risk seemed to be higher in the lower extremity than at other sites. Others have also identified an association between T1DM and fractures. A cross-sectional case-control study of 82 young eastern European participants (mean age 31.1) with T1DM revealed a 320% increased risk of asymptomatic vertebral fractures compared with controls which was independent of multiple covariates, including lumbar spine bone density [16]. Similarly, a large registry based cohort study from Taiwan age and sex matched 500,868 diabetic patients (identified by diagnosis codes) and found that men and women with diabetes had a 28% and 72% increased risk of hip fracture compared to controls without diabetes. When stratified by age this study revealed that the hazard ratios (HR) for hip fracture were higher for diabetic men and women aged 35 to 44 and were null statistically in men over 74 and women over 84 [17]. It may be that age or menopause related changes overshadow the effect of diabetes on fracture risk, which is why we see disparities between the young and old.

Several studies have found associations between T2DM and skeletal fractures. In the Women’s Health Initiative observational cohort 93,676 generally healthy postmenopausal women were followed for 7 years. Women with T2DM at baseline had a 20% increased risk of fracture at any site after adjusting for multiple covariates, including frequency of falls [9]. The Study of Osteoporotic fractures followed 9654 women 65 years or older for an average of 9.4 years. Radiology reports were used to confirm reported incident fractures. They found that women diagnosed with diabetes after the age of 40 had a 30% increased risk of having any non-vertebral fracture and an 82% increased hip fracture risk compared with those without diabetes. The risk of vertebral fracture was not significantly increased in this analysis [18]. Other studies have specifically evaluated vertebral fractures. A cross-sectional study from Japan ascertained fractures from spinal and thoracic radiographs in participants over 50 years old and found that men and women with T2DM had a 373% and 82% increased risk of having prevalent vertebral fractures respectively after adjustment for lumbar spine bone density [19]. In contrast, a cross-sectional evaluation of the Canadian Multicenter Osteoporosis study did not find that self-reported T1DM nor T2DM was associated with vertebral deformity determined by spinal radiographs among older men and women (mean age 66) [20]. One population-based study evaluated all reported fractures in Denmark and found that T2DM was associated with a 19% increased risk of any fracture while T1DM was associated with 30% increased risk. In this study T1DM was associated with a significantly increased risk of spine fracture while T2DM was not, in contrast both T1DM and T2DM were associated with increased risk for hip fracture when compared to age and sex matched controls [21].

Data from these large well executed studies across multiple populations consistently find that people with T1DM or T2DM have increased fracture risk. Findings for hip fractures seem to be more consistent than those for vertebral fractures, but may depend on the specific populations studied. T1DM may be associated with a lifelong increased risk of fracture. Two meta-analyses evaluating published case-control and cohort studies have found that hip fracture risk was significantly higher for T1DM than T2DM without overlapping confidence intervals [22, 23], suggesting the magnitude of fracture increase is higher in T1DM than in T2DM. It may be that there are different mechanisms of increased fragility for T1DM and T2DM or that disease duration and diabetes control have modulating effects. A summary of fracture risk by diabetes type is presented in Table 1.

**Table 1:** Association of diabetes mellitus and fracture risk

Type of diabetes	Fracture type	Findings	Study design	Reference
Type 1 diabetes mellitus	Hip fracture	**RR 7.10 (CI 95%: 4.4–11.4)**	Prospective observational	Janghorbani et al.
Type 1 diabetes mellitus Men 60-69yo	Any type	**HR 2.00 (CI 95%: 1.63–2.45)**	Prospective observational	Weber et al.
	Hip fracture	**HR 5.21 (CI 95%: 3.2–8.47)**		
Type 1 diabetes mellitus Men 40-49yo	Any type	**HR 1.82 (CI 95%: 1.53–11.43)**		
Type 1 diabetes mellitus Men 30-39yo	Hip fracture	**HR 4.16 (CI 95%: 1.52–11.43)**		
Type 1 diabetes mellitus	Vertebral fracture	**OR 4.20 (CI 95%: 1.40–12.7)**	Cross sectional	Zhukouskaya et al.
Type 1 diabetes mellitus men	Hip fracture	**HR 1.28 (CI 95%: 1.21–1.34)**	Prospective observational	Chen et al.
Type 1 diabetes mellitus women	Hip fracture	**HR 1.72 (CI 95%: 1.66–1.78)**		
Type 1 diabetes mellitus	Hip fracture	**RR 6.94 (CI 95%: 3.25–14.78)**	Meta-analysis observational studies	Vastergaard et al.
Type 2 diabetes mellitus	Hip fracture	**RR 1.38 (CI 95%: 1.25–1.53)**		
Type 2 diabetes mellitus	Any site	**RR 1.20 (CI 95%: 1.11–1.30)**	Prospective observational	Bonds et al.
Type 2 diabetes mellitus	Non-vertebral	**RR 1.30 (CI 95%: 1.10–1.53)**	Prospective observational	Schwartz et al.
	Hip fracture	**RR 1.82 (CI 95%: 1.24–2.69)**		
	Vertebral	RR 1.12 (CI 95%: 0.69–1.83)		
Type 2 diabetes mellitus Men	Vertebral	**OR 4.73 (CI 95% 2.19–10.20)**	Cross sectional	Yamamoto et al.
Type 2 diabetes mellitus Women	Vertebral	**OR 1.86 (CI 95% 1.11–3.12)**		
Type 2 diabetes mellitus Men	Vertebral	OR 0.77 (CI 95% 0.48–1.22)	Cross sectional	Hanley et al.
Type 2 diabetes mellitus Women	Vertebral	OR 0.92 (CI 95% 0.67–1.26)		

Findings in bold letters indicate a significant positive association

*RR*: relative risk; *HR*: hazard ratio; *OR*: odds ratio

### Diabetes and bone density

In T1DM, the association of bone mineral density with increased fracture risk is one of the most studied potential mechanisms. Bone density is decreased in T1DM. In a study of premenopausal women, those with T1DM had lower total hip (TH), femoral neck (FN) and whole body BMD after adjusting for multiple covariates, with no difference in lumbar spine (LS) BMD. The bone turnover markers osteocalcin and N-telopeptides of type I collagen were evaluated but did not significantly change the diabetes-BMD association [24]. In one small European study men with T1DM (mean age 43) had similar TH BMD but significantly lower spine BMD z score than age matched controls (Z score − 0.705 vs. -0.099) [25]. In the Fremantle diabetes study in Australia middle aged men with T1DM had significantly lower TH and FN BMD when compared to age and sex matched controls, but women with T1DM and matched controls had similar BMD at each site [26]. Multiple other studies have found lower BMD in T1DM, often associated with the presence of microvascular complications [24, 27–30]. The limited sample size in these individual studies preclude wide generalizations, however a systematic review and meta-analysis found that aggregate estimates of published studies showed significantly lower BMD at the spine (Z score − 0.22 ± 0.01) and hip (Z score − 0.37 ± 0.16) in participants with T1DM compared with those without diabetes [22].

In children, low bone density for age may be present early after diagnosis of T1DM. A case control study evaluating children with recently diagnosed T1DM revealed significantly decreased LS BMD and decreased bone formation markers when compared to age, height, and pubertal status matched controls [31]. This study also revealed that LS BMD was significantly lower in those with longer duration since T1DM diagnosis, indicating that diabetes may hamper acquisition of peak bone density during development. Similarly, a cross sectional study conducted in Caucasian children and adolescents with and without T1DM related complications found that duration of diabetes in years was negatively associated with both LS and total body BMD [32]. Bone turnover markers were negatively associated with hemoglobin A1C (HgbA1C) in this study, indicating a potential beneficial role of improved glucose control on bone growth. An additional case control study conducted on 86 younger participants (mean age 27.2) with T1DM, BMD at the total body and LS sites were significantly decreased compared to controls [33]. These findings must be interpreted with caution however, since children with chronic illnesses may have delayed puberty and therefore may not have the same bone size as controls, a factor which could lead to the appearance of significantly lower BMD [34].

In T2DM many studies have not found decreased BMD and some have shown paradoxically increased BMD. In the Women’s Health Initiative women with T2DM had statistically significant increases in BMD at the spine and hip compared to women without diabetes throughout 9 years of follow up [9]. Other studies have shown increased BMD at the lumbar spine and hip in men and women with T2DM who were not using insulin [35], and increased BMD at the hip and forearm in women with T2DM [26]. One meta-analysis found that in T2DM a composite Z score was 0.41 higher at the spine and 0.27 at the hip than in non-diabetic controls. The same study performed a meta-regression analysis and found that BMI was a predictor of bone density in T2DM but not T1DM [22]. Increased BMI has well established associations with development of T2DM [36–39] and weight is also associated with increased bone density at weight bearing sites [40–42]. However some studies have shown that fracture risk in T2DM is independent of BMI or weight and height [9, 18], and BMD [18, 19, 43] which may indicate that the combined adverse effects of T2DM on bone may overwhelm any of the potential protective benefits from increased bone density.

One analysis of the osteoporotic fractures in men study evaluated volumetric bone mineral density (vBMD) and estimated bone strength using polar strength strain index and section modulus derived from peripheral quantitative computed tomography (pQCT). Older men with T2DM had bone strength that is low despite no difference in cortical vBMD [44], a finding that could imply that normal BMD should not be considered clinically reassuring in diabetes. Other studies have found that the presence of microvascular disease was associated with deficits in microarchitecture of bone, specifically of cortical and trabecular vBMD in T1DM [45] and cortical bone in T2DM [46], which may be driven by high cortical porosity [47]. These structural changes could partly explain the excess fracture risk in these populations.

T1DM and T2DM appear to interact differently with BMD. T1DM may contribute to low BMD, perhaps due to younger age at onset affecting growth of bone and peak bone mass. Increased weight and BMI, a common pathway for both increased BMD and development of T2DM may account for the increased BMD in T2DM. High BMD in T2DM is not entirely protective however, and bone strength may actually be lower than what is predicted for BMD. The microvascular changes of diabetes have been associated with microarchitectural bone defects, which may lead to bone fragility.

### Mechanisms for increased fracture risk in diabetes

Shared mechanisms for increased fracture risk in both T1DM and T2DM include accumulation of advanced glycation end-products (AGEs) [48–51], chronic hyperglycemia, poor blood glucose control [43, 52], hypercalciuria [53] and high propensity for falls [54, 55]. AGEs are permanently deposited glyco-oxidation products whose formation is thought to be stimulated by intracellular hyperglycemia [56]. AGEs can form cross-links with proteins like collagen that affect their structure and functions [49]. A growing body of evidence indicates that AGEs play a crucial role in the progression of classical diabetes complications [57] and diabetic osteopathy. Collagen is a prominent component of bone and when AGEs such as pentosidine and carboxymethyl lysine are produced in collagen fibers, bone strength deteriorates [58–61], which is one potential explanation for why the increased fracture risk in T1DM and T2DM appears to be independent of BMD [51, 62, 63]. Elevated glucose levels accelerate AGE formation [64] and so diabetes control could be an important determinant of bone fragility. Hyperglycemia has direct effects on bone cells as well, inhibiting osteoclastogenesis [65].

Diabetes is associated with decreased bone turnover which could have deleterious effects on bone health. Rodent models of insulin deficiency can have decreased bone growth and turnover driven by decreased osteoblast recruitment. This phenotype can be partially corrected by administration of insulin like growth factor-1 (IGF-1) [66]. In T1DM and in the late stages of T2DM, insulin deficiency could impair bone homeostasis through dysregulation of the growth hormone-IGF-1 axis [53]. Pooled data has shown that bone formation markers such as osteocalcin, procollagen type 1 amino terminal pro-peptide and bone resorption markers such as C-terminal cross-linked telopeptide were significantly lower among those with diabetes and did not necessarily correlate with glucose level [67]. It has been suggested that diabetes mellitus should be considered a state of low bone turnover, perhaps driven by increased serum levels of sclerostin and osteoprotegerin which are known to inhibit osteoblast and osteoclast differentiation respectively [68].

The microvascular complications of diabetes mellitus have been associated with an increased propensity for falls, which could partially explain increases in skeletal fractures. In a cohort of well-functioning older adults, diabetes-related complications of reduced peripheral nerve function, poor vision, and decreased renal function were all associated with increased risk of falls. The study further suggested that even a modest decline of renal function could account for increased fall risk through lower muscle strength and nerve function from lower levels of active vitamin D [54]. The Study of Osteoporotic Fractures revealed an increased risk of falls in women with diabetes, especially in those treated with insulin, who had more than double the risk of having multiple falls than women without diabetes [55] . Poor balance and peripheral neuropathy were found to be important risk factors associated with falls in this study.

### Glucose control, length of disease and fracture risk

Given the potential mechanisms described above one would expect that the higher glucose levels from poor diabetes control and longer exposure to diabetes may result in increased fracture risk. This principle has been borne out in several studies. The Action to Control Cardiovascular Risk in Diabetes (ACCORD) trial randomized participants with T2DM to intensive or standard glycemic control strategies. Those in the intensive glycemia group achieved a median HgbA1C of 6.4% as compared to 7.5% in the standard glycemia group. They found that intensive glycemic control was not significantly associated with fracture or fall risk compared with standard therapy [69]. In the Health in Aging and Body Composition prospective cohort study adults 70–79 years old with T2DM (HgbA1C > 7%) were found to have a 64% increased risk of incident clinical fractures while those with Impaired Fasting Glucose, an intermediate state of abnormal glucose metabolism between normal glucose homeostasis and diabetes, did not have significantly increased risk. In this study participants with T2DM had reduced peripheral sensation, lower lean mass, more falls and lower total hip bone density, highlighting diabetic complications as important considerations in the elderly individuals with T2DM [70]. A community-based prospective cohort study stratified participants with known diabetes by HgbA1C level and compared the risk of hospitalization due to fracture across a median follow up of 20 years. Participants with HgbA1c ≥8% had a 63% higher risk of hospitalization due to fracture compared with those whose HgbA1C was under 8% [71]. Those with unrecognized diabetes prior to study initiation and pre-diabetes did not have increased hospitalization risk compared to those with HgbA1C under 5.7%. Similarly, a prospective population-based cohort in the Netherlands determined that patients with T2DM and HgbA1C over 7.5% had a 62% increased risk of all types of fractures compared to those with HgbA1C under 7.5% after adjustment for covariates including FN BMD [43]. In the United States (US) NHANES database those with diagnosed diabetes or HgbA1C above 6.5% had a significantly increased risk of non-skull fractures compared to non-diabetes controls but those with pre-diabetes did not have a significantly increased fracture risk [72]. Epidemiological data from the National Diabetes Care Program in Taiwan revealed that among 20,025 patients with T2DM aged 65 years or older the risk of hip fracture appeared to increase in a dose-response relationship with each 1% HgbA1C increase above 8%, compared to those with HgbA1C of 6–7%. The increased hip fracture risk was maintained after adjustment for co-variates among patients with HgbA1C levels in the 9–10% range and those with HgbA1C above 10%, suggesting that fracture risk may be increased commensurate to the magnitude of poor glucose control [52]. Similar findings were noted in patients with T1DM in the THIN study where each 1% greater average HgbA1C level was associated with a 5% greater risk of incident fractures in males and 11% greater risk in females [15]. In this study, diabetic neuropathy and retinopathy were found to be risk factors for fracture in males but only diabetic neuropathy was significant in females.

Longer duration of diabetes appears to increase fracture risk as well. In the Nurses’ Health study there was a significant trend for greater fracture risk with increased duration of diabetes. Fracture risk was increased by 200% with diabetes duration over 12 years [14]. In a cohort of 82,094 diabetic adults in Manitoba, Canada diabetes duration of over 5 years increased the risk of combined hip, wrist and spine fractures compared to age and sex matched controls. Interestingly, newly diagnosed diabetes was found to significantly reduce the risk of fracture in this cohort [73]. In a prospective study of the residents of Blue Mountains in Australia diabetes duration over 10 years was also significantly associated with all fractures [74].

Poor glucose control and longer exposure to hyperglycemia are known to lead to increased AGEs and the development of the microvascular complications of diabetes including retinopathy, neuropathy and nephropathy [56, 75, 76] which have been associated with microarchitectural changes in bone as previously discussed. These mechanisms may be leading to bone fragility. More data from large prospective cohorts are needed to evaluate the direct effects of microvascular complications on fracture risk. It seems that fracture risk is lower in patients with HgbA1C under 7.5% and may increase as HgbA1C climbs over 8%. However, it appears that improvement beyond reasonable control (HgbA1C around 7.5%) may not lead to additional benefit. While initially T2DM may be protective for fracture, possibly due to hyperinsulinemia through insulin’s homology with IGF-1 causing increased bone strength [53, 77], longer exposure of diabetes is associated with increased fracture risk.

### Glucose-lowering medications and fracture risk

Medications used to treat diabetes mellitus can modulate fracture risk. Treatment with thiazolidinediones increased risk of fractures in women with T2DM independent of age and duration of exposure [78, 79]. Thiazolidinediones activate peroxisome proliferator-activated receptors (PPARs) which are factors that promote adipogenesis. Mesenchymal stem cells (MSC) are the common precursors of adipocytes and osteoblasts and PPARγ is an important regulator of MSC differentiation [80]. The increased fracture risk with thiazolidinediones could be due to activation of PPARγ shifting differentiation of MSCs towards adipogenesis and away from osteogenesis through the suppression of key osteogenic transcription factors. Insulin and sulfonylurea use has also been associated with increased fracture risk [81, 82] a finding which could be partially explained by the higher incidence of hypoglycemic events and risk of falls [54, 82]. Metformin has been shown to have a positive or neutral effect on BMD and fracture risk [21]. Sodium glucose co-transporter-2 (SGLT-2) inhibitors such as dapaglifozin did not impact bone turnover markers or BMD [83]. Canaglifozin has been associated with bone loss and increased fracture risk at the hip [84]. More studies are needed to clarify the SGLT-2 class effect on bone fracture risk. Clinical evidence is lacking for dipeptidyl peptidase-4 inhibitors and glucagon like peptide-1 analogs [85].

### Fracture risk stratification for diabetes

FRAX is a useful but imperfect tool for fracture risk stratification [86]. Osteoporosis guidelines in the US and UK recommend its use in treatment algorithms [2, 87, 88]. FRAX has been shown to underestimate major osteoporotic fracture and hip fracture risk in patients with diabetes [10, 89]. The University of Manitoba group has made great contributions in determining the effects of diabetes on fracture risk stratification with FRAX. They have shown that the effect of diabetes is independent of other FRAX risk factors, but appears to be more important for hip fracture prediction in younger individuals [90]. Duration of diabetes was associated with hip fracture independent of FRAX scores in a dose dependent fashion, but was associated with increased major osteoporotic fracture risk only at 10 years duration [91]. One strategy to modify FRAX scores to more accurately reflect the estimated fracture risk in diabetes is to include rheumatoid arthritis as a proxy for diabetes since they have similar effects on the FRAX algorithm [92]. Trabecular bone score (TBS) is a technology that when applied to bone density by DXA in the lumbar spine, predicts fractures [93] and identifies a greater proportion of those at risk than BMD in T2DM [94]. TBS can be used with FRAX to improve fracture prediction [95] and may be useful in T2DM, but is not commonly available in many clinical practices.

## Conclusions

Type 1 and Type 2 diabetes mellitus both increase the risk of skeletal fracture, particularly at the hip. The etiology of diabetes determines its effects on BMD. Type 1 diabetes is associated with BMD decrease while T2DM is associated with normal to increased BMD. T1DM and T2DM have common mechanisms such as AGEs deposition and bone microarchitectural defects where cortical bone appears to be particularly affected. The increased fracture risk in DM is independent of FRAX and must be considered when risk stratifying patients in clinical practice. Diabetes mellitus should be considered an important fracture risk factor.

## Abbreviations

AGEs: Advanced glycation end products
BMD: Bone mineral density
FN: Femoral neck
HgbA1C: Hemoglobin A1C
HR: Hazard ratio
IGF-1: Insulin like growth factor-1
LS: Lumbar spine
PPAR: Peroxisome proliferator-activated receptors
SGLT-2: Sodium glucose co-transporter-2
T1DM: Type 1 diabetes mellitus
T2DM: Type 2 diabetes mellitus
TH: Total hip
THIN: The Health Improvement Network
vBMD: Volumetric bone mineral density
MSC: Mesenchymal stem cells
TBS: Trabecular bone score
US: United States
UK: United Kingdom
